# Supplementary material for: Changes in transmission of Enterovirus D68 (EV-D68) in England inferred from seroprevalence data
Source: eLife. 2023 Jun 9;12:e76609. doi: 10.7554/eLife.76609 (PMC10259368; doi:10.7554/eLife.76609)
Supplement: Supplementary file 1. — (a) Proportion of seropositive individuals in the 25–40 years old group. This proportion is used to fix the proportion of individuals born with maternal antibodies and is assumed constant across the study. (b) Model comparison for the two different datasets, corresponding to two different seropositivity cut-offs. For each model comparison, the first row corresponds to the model with the largest expected log pointwise density (ELPD), which measures the model expected predictive accuracy. For each model, its log-likelihood and the difference in the Bayesian leave-one-out (LOO) estimate of the ELPD compared to the best model are shown. (c) Model parameter estimates. Median and 95% credible intervals are presented. [file elife-76609-supp1.docx]

**Supplementary file 1: Supplementary results**

**Supplementary file 1a.** Proportion of seropositive individuals in the 25-40 years old group. This proportion is used to fix the proportion of individuals born with maternal antibodies and is assumed constant across the study.

| Serosurvey | 1:16 cut-off | 1:64 cut-off |
| --- | --- | --- |
| 2006 | 0.987 | 0.882 |
| 2011 | 0.982 | 0.909 |
| 2017 | 0.977 | 0.920 |
| All years combined | 0.982 | 0.904 |

**Supplementary file 1b.** Model comparison for the two different datasets, corresponding to two different seropositivity cut-offs. For each model comparison, the first row corresponds to the model with the largest expected log pointwise density (ELPD), which measures the model expected predictive accuracy. For each model, its log-likelihood and the difference in the Bayesian leave-one-out (LOO) estimate of the ELPD compared to the best model are shown.

|  | | log-likelihood | | ΔLOO | |
| --- | --- | --- | --- | --- | --- |
| *Cut-off 1:16* | | | | |  |
| Model 2 | -130.93 | | 0.00 | |  |
| Model 1 | -154.29 | | -21.77 | |  |
| *Cut-off 1:64* | | | | |  |
| Model 2 | -204.66 | | 0.00 | |  |
| Model 1 | -258.20 | | -53.10 | |  |

**Supplementary file 1c.** Model parameter estimates. Median and 95% credible intervals are presented.

|  | Cut-off 1:16 | Cut-off 1:64 |
| --- | --- | --- |
| *Model 1* | | |
| $\boldsymbol{\omega}$ | 0.227 (0.181-0.282) | 1.911 (1.320-2.778) |
| $\boldsymbol{\lambda}$ | 0.521 (0.421-0.648) | 0.109 (0.100-0.118) |
| *Model 2* | | |
| $\boldsymbol{\omega}$ | 0.682 (0.448-1.045) | 2.442 (1.661-3.678) |
| $\boldsymbol{\lambda}_{\boldsymbol{2017}}$ | 0.739 (0.547-0.991) | 0.221 (0.155-0.296) |
| $\boldsymbol{\sigma}$ | 0.062 (0.037-0.111) | 0.023 (0.013-0.039) |
